# Xuedan Sustained Release Pellets Ameliorate Dextran Sulfate Sodium–Induced Ulcerative Colitis in Rats by Targeting Gut Microbiota and MAPK Signaling Pathways

**DOI:** 10.3389/fphar.2022.833972

**Published:** 2022-04-20

**Authors:** Yingchun Zhang, Dan Feng, Yue Zeng, Hanyu Zhang, Xiaohong Du, Yang Fu, Xinhui Wang, Dingyue Lian, Ruikang Wang, Hongyu Xiao, Ning Wei, Fuqiang Zhai, Hanru Liu

**Affiliations:** ^1^ College of Pharmaceutical Sciences and Chinese Medicine, Southwest University, Chongqing, China; ^2^ Research Institute for New Materials and Technology, Chongqing University of Arts and Sciences, Chongqing, China

**Keywords:** Cucurbitacins, ulcerative colitis, short-chain fatty acids, gut microbiota, MAPK

## Abstract

Cucurbitacins have a variety of bioactivities, such as anticancer, anti-inflammatory, antidepressant-like, and antiviral effects, but their pharmacological effect in ulcerative colitis (UC) has not been reported until now. Thus, this study aims to investigate the preventive effects of Xuedan sustained release pellets (XSPs) on UC rats and the underlying mechanisms. XSPs were prepared by extracting cucurbitacins from *Hemsleya*. Experimental UC rats were induced by the intake of 4% dextran sulfate sodium (DSS) for a week and treated with different doses of XSP (0.95, 1.90, and 3.8 mg/kg). The body weight, colon length, disease activity index (DAI), and histological changes of colonic tissue were measured. In addition, the expressions of pro-inflammatory cytokines were detected by using the enzyme-linked immunosorbent assay. Pathways involved in the intestinal inflammation were targeted by RNA-sequencing. Moreover, the changes of gut microbial diversity and composition were analyzed by the 16SrNA analysis and the contents of short-chain fatty acids (SCFAs) were detected by GC-MS. The results revealed that XSP intervention greatly restored the weight loss and colonic shortening (*p* < 0.05) and reduced the raised DAI scores, myeloperoxidase, and nitric oxide activities in UC in rats (*p* < 0.05). XSP administration also downregulated the protein levels of pro-inflammatory factors IL-1β, IL-6, and TNF-α. Notably, it was found that XSP considerably suppressed the activation of the MAPK signaling pathway. In addition, XSP treatment improved the balance of gut microbiota that was disturbed by DSS. The beneficial bacteria Lachnospiraceae_NK4A136 group and *Lactobacillus* at the genus level significantly increased in the XSP group, which had decreased with the use of DSS (*p* < 0.05). Pathogenic bacteria including *Escherichia–Shigella* and *Bacteroides* in UC in rats were reduced by XSP intervention. Furthermore, XSP significantly elevated the production of SCFAs in UC in rats (*p* < 0.05). These alterations in inflammatory status were accompanied with changes in gut microbiota diversity and SCFA production. In conclusion, XSP exhibited protective effects against DSS-induced UC in rats. XSP treatment decreased inflammation *via* modulation of gut microbiota composition and SCFA production.

## Introduction

Ulcerative colitis (UC) is a chronic inflammatory disorder in the rectal and colonic mucosa (Ordás et al., 2012). The prevalence of UC is rising worldwide because of increasing incidence and low cure rate (Feng et al., 2021). Moreover, UC patients have an increasing risk of developing colorectal cancer (Eaden et al., 2001). Many factors, such as immunological deficiency, genetic context, and environmental factors, are considered the main causes of UC (Dutta and Chacko, 2016; Cho et al., 2017). However, the precise mechanisms of UC have not yet been completely elucidated. Drugs for treating UC are mainly immunosuppressive, anti-inflammatory, biological agents, and antibiotics (Bonovas et al., 2018; Bhattacharya and Osterman, 2020; Fiorino et al., 2021). These therapies can partly ameliorate clinical symptoms in UC patients. Nevertheless, side effects always occur when using these drugs (Armuzzi and Liguori, 2021). Therefore, it is necessary to find newer, safer therapies for UC patients.

Gut microbiota have received increasing attention for their crucial role in maintaining a healthy host. A large number of studies have reported that gut microbiome disorders coincide with a variety of human diseases. These diseases include UC, colon cancer (Kinross et al., 2008; Young et al., 2010; Dickson, 2017), obesity (Turnbaugh and Gordon, 2009; Sweeney and Morton, 2013), diabetes (Brown et al., 2011; Devaraj et al., 2013), central nervous system disorders (Ghaisas et al., 2016), and cardiovascular diseases (Kelly et al., 2016; Saji et al., 2019). The main metabolic products, short-chain fatty acids (SCFAs), are produced by gut microbiota by fermenting undigested complex carbohydrates in the colon (Rooks and Garrett, 2016).

Accumulating evidence reveals that SCFAs play an essential role in maintaining the balance between gut flora and the host immune system (Fattahi et al., 2020; Campos-Perez and Martinez-Lopez, 2021; Roopashree et al., 2021), resulting in SCFAs being recognized as crucial factors for health protection by promoting metabolism and immune homeostasis (Liu et al., 2021; Mallick and BasakDuttaroy, 2021; Salsinha et al., 2021). SCFAs are small-molecule metabolites and consist mainly of acetate, propionate, and butyrate (Koh et al., 2016). Previous reports have shown that SCFAs could decrease inflammation by modulating p38 MAPK, NLRP3, and MEK–ERK signaling pathways (Segain et al., 2000; Park et al., 2007; Yonezawa et al., 2007; Kobayashi et al., 2017). Many factors regulate SCFA production, including bacterial communities in the gut, aging, and dietary fibers (Macfarlane and Macfarlane, 2003; Ran An et al., 2019; Mizuno et al., 2020). Treatment with exogenous SCFAs and increasing endogenous SCFAs have been proposed to reduce inflammation in UC (Vernia, 2007; Couto et al., 2020; Morales Fénero et al., 2021; Xiao et al., 2021). Among various therapies, herbal medicine has exhibited potential anti-inflammatory effects by regulating gut microbiota diversity and SCFA production (Feng et al., 2018; Xuedong An et al., 2019). Therefore, it can be considered an alternative treatment method for UC (Lin et al., 2020; Fan et al., 2021).

Xuedan is the dry root tuber of the *Hemsleya* genus in the Cucurbitaceae family. The medicinal plants are mainly distributed in Chongqing, Sichuan, Yunnan, and Guizhou provinces (Ling Xu Li et al., 2016). Xuedan possesses the traditional functions of clearing heat and eliminating toxins and relieving swelling and pain. The crude drug is traditionally used as a remedy for bacillary dysentery and gastroenteritis (Yang and Mo, 2004). A recent research study has revealed that Xuedan is rich in various active substances, including cucurbitacins, glycosides, lignans, phenolics, and other constituents (Kubo et al., 1996; Li et al., 2015; Song et al., 2015; Ye-Dan Li et al., 2016; Li et al., 2017; Jin et al., 2019). Recent studies have also shown that Xuedan has anticancer, anti-HIV, and anti-inflammatory activities, in which cucurbitacins play a crucial role in curing related diseases (Wu et al., 2002; Tian et al., 2008; Peng et al., 2020; Kim et al., 2015). Cucurbitacin preparations, such as Xuedan tablets, are clinically used for treatment of respiratory and digestive tract inflammation. Thus, because of the anti-inflammatory effects of cucurbitacins in other tissues, we hypothesized that these cucurbitacin ingredients might be effective in modulating colonic inflammation. In the present study, we aimed to investigate the possible preventive effects of XSP on DSS-induced UC in rats and uncover the possible mechanisms.

## Materials and Methods

### Materials and Reagents

The crude form of Xuedan was bought from the Qiancaoyuan Chinese Medicinal Materials Management Department (Kunming, China). A voucher specimen was deposited at the College of Pharmaceutical Sciences and Chinese Medicine, Southwest University. Standard cucurbitacin IIa (purity >98%) was bought from the National Institute for the Control of Pharmaceutical and Biological Products (Beijing, China). Dextran sodium sulfate (DSS) was obtained from Meilunbio (MW:36000-50000, Dalian, China); the fecal occult blood test kit was supplied by the Nanjing Jiancheng Bioengineering Institute (Nanjing, China); enzyme-linked immunosorbent assay (ELISA) kits for IL-1β, TNF-α, IL-6, IL-10, nitric oxide (NO), and myeloperoxidase (MPO) analyses were purchased from Abebio (Wuhan, China). High-performance liquid chromatography (HPLC)–grade acetonitrile was purchased from Tedia Company, Inc. (Fairfield, OH, United States), and other chemical regents were of analytical purity.

### Preparation of Xuedan Sustained Release Pellets

Xuedan was ground to powder and refluxed for 2 h in 10 times its volume of 95% ethanol at 80°C. The extraction was repeated three times. Subsequently, the extracted solutions were combined and filtered to discard the remains. Next, the filtrate was condensed by a rotary evaporator. Then, petroleum ether was added to degrease the concentrated solutions. After that, cucurbitacins were extracted from the purified solutions by using ethyl acetate. Finally, the extracted solutions were concentrated *via* the rotary evaporator and dried in a vacuum oven for 48 h. The solid residue was collected and used as the pellet cores of XSPs, and then the pellet cores were prepared by extrusion–spheronization technology. Fluidized bed coating technology was used to coat the pellet cores. The morphological traits of the coating layer and pellets were observed by scanning electron microscopy (SEM, Zeiss Sigma HD). The physiochemical properties of XSPs were also determined. In addition, the *in vitro* release rate was detected according to general principles of the Pharmacopoeia of the People’s Republic of China (2020 version). The release of the drug was quantified by HPLC.

Before pharmacological evaluation, the main phytochemical component, cucurbitacin IIa, in XSP was analyzed and quantified by HPLC. Briefly, a Shimadzu LC-20A instrument was used for HPLC analysis. The separation was performed on an Ultimate XB-C_18_ (250 mm × 4.6 mm; 5 μM, Welch Material, West Haven, CT, United States). The eluent solvents consist of deionized water (A) and acetonitrile (B) in a constant proportion of 65:35 and flow rate of 1.0 ml/min; 10 µl of the sample solution was used for HPLC analysis. The ultraviolet spectrum was set at 202 nm for producing chromatograms of cucurbitacin IIa. The HPLC method showed good linearity, precision, repeatability, and stability under the aforementioned analytic conditions.

### Experimental Animals and Treatment

All adult Sprague–Dawley (SD) rats (180–230 g; certificate number SCXK (Xiang) 2016-0002) were provided by Hunan SJA laboratory Animal Co., Ltd. and maintained in appropriate conditions (22 ± 1°C and 12-h light/dark cycle). After several days of acclimatization, the rats were randomly divided into five groups (*n* = 10 per group) as follows: the control group; DSS group (4% DSS); LXSP group (4% DSS + 0.95 mg/kg XSP); MXSP group (4% DSS +1.90 mg/kg XSP); and HXSP group (4% DSS + 3.80 mg/kg XSP). The rats were administered 4% DSS in drinking water over a period of 7 days to induce UC (Xu et al., 2021; Zhang et al., 2021). The animals in the XSP groups were continuously administered XSP *via* gavage for 10 days. The rat body weight, stool consistency, and stool occult blood tests were regularly measured to assess UC severity during the experimental period. The animals were killed at day 11 of the experiment. The blood samples were harvested from the abdominal aorta and immediately centrifuged at 3,000 × g for 15 min at 4°C. The pellet was discarded, and the supernatant was collected for further analysis. Colonic tissues were removed and washed with ice-cold phosphate-buffered saline (PBS). Subsequently, the length and weight of the colons were determined. A portion of the colon was immediately fixed in 4% paraformaldehyde overnight. Thereafter, the tissue was embedded in paraffin for histological inflammation assessment. The remaining samples were stored at −80°C for further analysis. All animal procedures in this study were performed according to the Guide for the Care and Use of Laboratory Animals. This study was also approved by the Animal Experiment Ethics Committee of Southwest University.

### Histologic Examination

The fixed colonic tissues were washed with deionized water for 30 min and dehydrated with 75, 85, 95, and 100% ethanol. Then, the tissues were soaked in xylene solution for permeabilization. Subsequently, the tissues were immersed into paraffin and cut into 5-μm sections. Hematoxylin/eosin (H&E) was used to stain these sections for histological examination. The changes in colon histopathology were visualized under a microscope. The pathological scores were calculated to evaluate the degree of colon injury.

### ELISA Analysis

The levels of inflammatory mediators (IL-1β, IL-10, IL-6, and TNF-α) in blood samples were measured using an ELISA kit, according to the manufacturer’s protocol. Meanwhile, the activities of MPO and NO were evaluated to reflect neutrophil infiltration into the inflamed colonic mucosa. MPO and NO were measured by using ELISA kits.

### Fecal 16SrNA Analysis

Fecal genomic DNA was extracted using the TGuide S96 Magnetic Stool DNA Kit (Tiangen Biotech Co., Ltd. Beijing, China), according to manufacturer’s instructions. The DNA quality was assessed using the Qubit dsDNA HS Assay Kit and Qubit 4.0 Fluorometer (Invitrogen, Oregon, United States). The special region (V3-V4) of the 16S rRNA gene in DNA samples was amplified using the general primers 338FP 5′-ACT​CCT​ACG​GGA​GGC​AGC​A-3′ and 806RP 5′-GGACTACHVGGGTWTCTAAT-3'. The reaction volume for the polymerase chain reaction (PCR) was 10 μl, including 338FP (10 μM) 0.3 μl, 806RP (10 μM) 0.3 μl, KOD FX Neo 0.2 μl, KOD FX Neo Buffer 5 μl, dNTP (2 μM) 2 μl, DNA template 25 ng, and distilled water up to 10 μl. The PCR products were further purified using Agencourt AMPure XP Beads (Beckman Coulter, Indianapolis, IN, United States) and quantified using the Qubit 4.0 Fluorometer (Invitrogen, Thermo Fisher Scientific, Oregon, United States). Then, the purified PCR products were mixed in equal amounts to construct a library. The library was sequenced on Illumina Novaseq 6000, and further bioinformatic analysis was carried out using BMKCloud (Biomarker Technologies Co., Ltd. Beijing, China).

### SCFA Measurement in Excrement

SCFAs were extracted from the fecal samples by using methanol solution. Briefly, 0.5 g of a fecal sample was suspended in 2 ml methanol, and the pH value was adjusted to 2.0 by sulfuric acid solution. Subsequently, the suspensions were placed in ice water for 20 min and instantly homogenized using a vortex mixer. The suspensions then were centrifuged at 12,000 × *g* for 15 min at 4°C. Finally, the supernatants were filtered and collected for further analysis. The contents of SCFAs were determined using a Shimadzu GC2010A (Kyoto, Japan) gas chromatography instrument coupled to a MS-QP2010 mass spectrometer. The inlet temperature was set at 220°C, and 1.0 μl of the sample was injected into the GC-MS system. The run time of the analysis was set to 17.5 min for each sample. The detection conditions were nitrogen gas set at a flow rate of 1.0 ml/min; ionization voltage, 70 eV; inlet temperature, 220°C; and detector temperature, 250°C. The total ion chromatograms were compared to the standard GS-MS chromatograms to identify the profiles of SCFAs according to peak similarities and m/z.

### RNA-Seq Analysis

Three group samples (control, DSS, and MXSP groups) were selected for RNA-seq analysis. Three biological repeats were performed for each group. TRIzol reagent (Invitrogen, United States) was used to extract total mRNA from the colon tissue according to the standard protocol. RNA quantity and purity were detected by using a Nanodrop 2000. The RNA integrity and renewable identification numbers (RIN) were verified using an Agilent 2100 Bioanalyzer. The mRNA sequencing library was constructed using an Ion Total RNA-Seq Kit v2 (Life Technologies, United States), according to the manufacturer’s instructions. Then, the cDNA libraries were sequenced using an Illumina HiSeq 2000. The following *de novo* assembly and bioinformatic analysis were performed on the Majorbio cloud platform (Majorbio, Shanghai, China). Differential expression genes (DEGs) of samples were determined using DEGseq (http://bioconductor.org/packages/stats/bioc/DEGSeq/), DESeq2 (http://bioconductor.org/packages/stats/bioc/DESeq2/), and edgeR (http://bioconductor.org/packages/stats/bioc/edgeR/).

### Statistical Analysis

Data were analyzed as mean ± standard deviation (SD). Statistical differences among the groups were measured by one-way analysis of variance (ANOVA) and multiple comparisons. A *p*-value <0.05 was set as statistically significant. The data calculations were carried out using GraphPad Prism 6.0 Software.

## Results

### Components and Release Profiles of XSP

**FIGURE 1:**
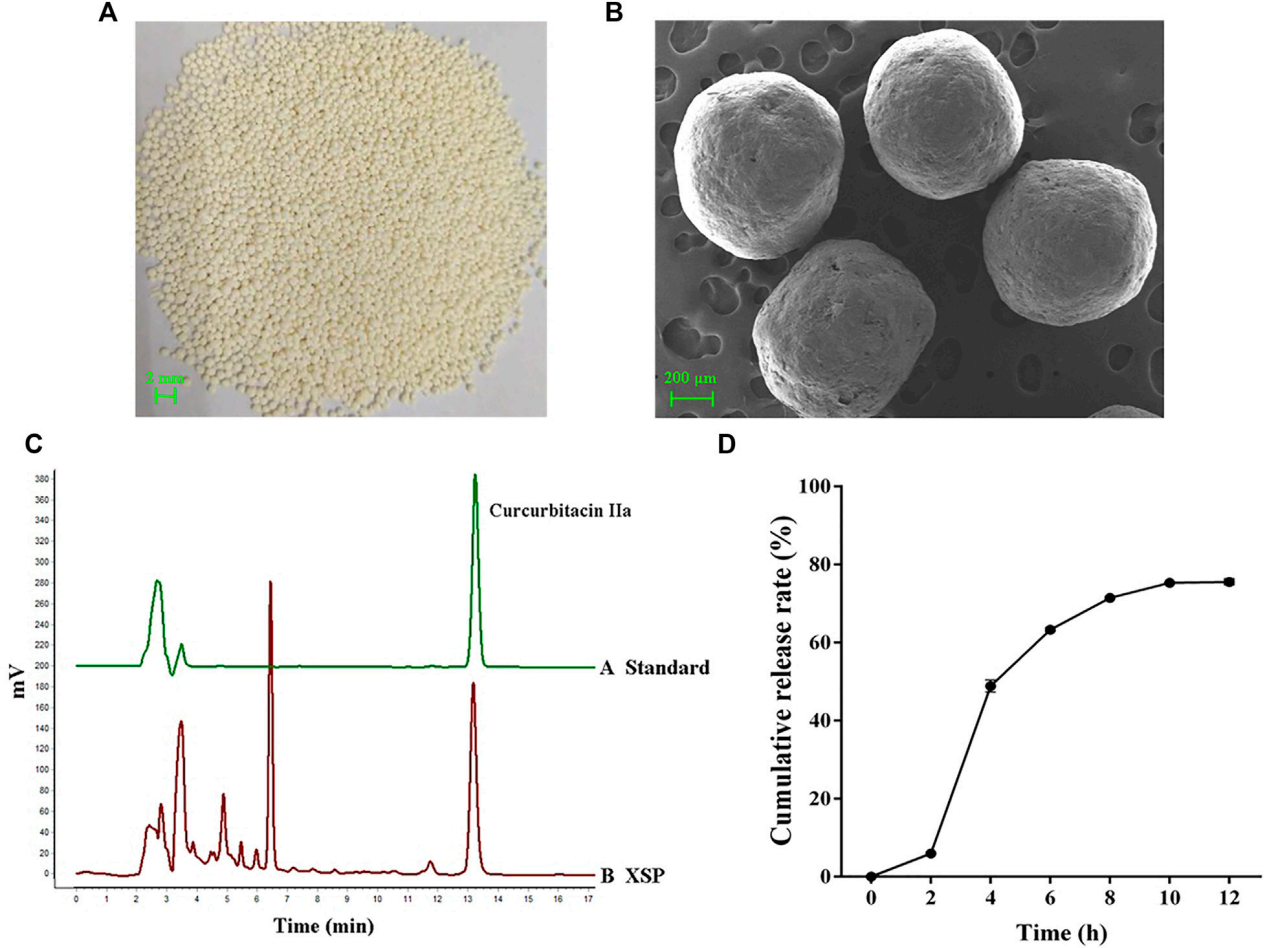
Quality evaluation of XSP. **(A)** Image showing the overall appearance traits of XSP. **(B)** SEM images showing the cross section of XSP. **(C)** HPLC properties of the representative component cucurbitacin IIa in XSP. **(D)** Graph showing the *in vitro* release of XSP at 0–12 h.

Among active components in XSPs, cucurbitacin IIa was identified as the major component. Cucurbitacin IIa accounts for 1.25% of Xuedan Materia Medica. After extraction and purification, the total collected cucurbitacins were used as the pellet core of XSPs. The surface of the pellets was scanned by SEM. It was observed that the pellets were spherical and had similar size. Moreover, the surface of pellets was coated compactly and had a round and smooth appearance. The bulk density and degree of roundness of XSP corresponded with the standards of the pellets. The content of cucurbitacin IIa was concentrated to 20.63% in XSPs and thus selected as the marker for the release rate of XSPs (Figure 1C). The drug release profiles in a simulated artificial intestinal environment were explored. Cucurbitacin IIa from XSPs was released in artificial intestinal fluid in a slow and sustained manner. To investigate the *in vitro* release performance of XSPs, the cumulative release of the XSPs was determined. There was little drug leakage in the artificial gastric fluid within 2 h, indicating that the pellets had an intact enteric coating layer. The cumulative release rate of the drug in artificial simulated intestinal fluid was more than 75% within the first 12 h. The result demonstrated that XSP has a constant release rate (Figure 1D).

### XSP Improves DSS-Induced Ulcerative Colitis in Rats

**FIGURE 2:**
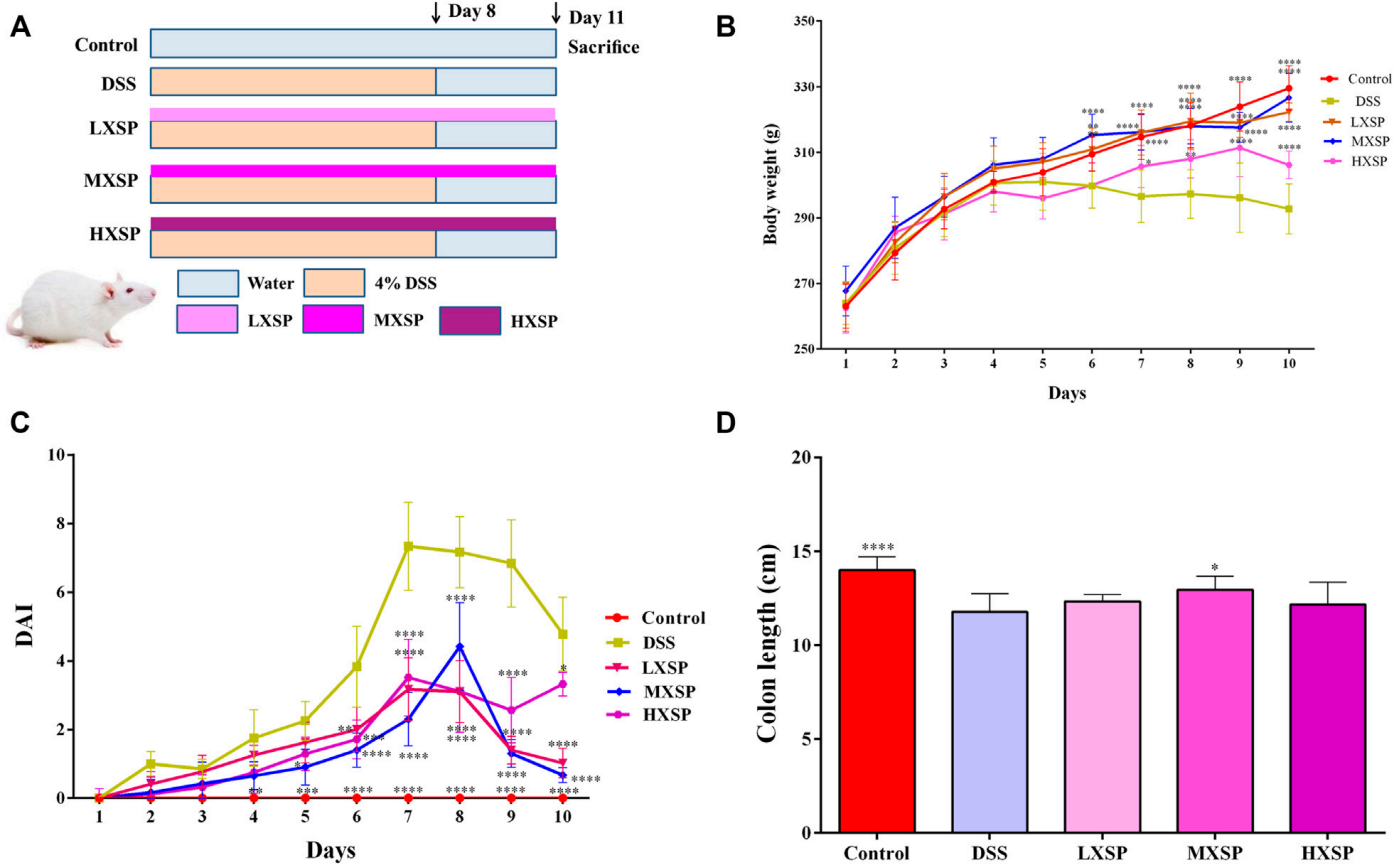
XSP treatment remarkably alleviates DSS-induced colitis in rats. **(A)** Flow chart of animal treatment (*n* = 10). **(B)** Changes of body weight (*n* = 6–10). **(C)** Disease activity index (DAI) scores (*n* = 6–10). **(D)** Colon lengths in different groups (*n*=6–10). Data were represented as mean ± SEM. *p < 0.05, **p < 0.01, ***p < 0.001, and ****p < 0.0001.

We administered 4% DSS to rats to induce the UC model. The body weight of rats was monitored daily. The body weight of animals in the DSS group began to decrease at day 5, while it gradually increased in other groups. Compared with the DSS group, animals treated with XSP exhibited a remarkable increase in body weight beginning at day 7 (Figure 2B; *p* < 0.05). The disease activity index (DAI) score increased distinctly in the DSS group compared to healthy controls from day 4 (Figure 2C; *p* < 0.01), whereas the DAI scores were markedly lower in the XSP groups with different doses (*p* < 0.05). Colonic shortening is another important symptom of colitis. As shown in Figure 2D, the colon length in the control group (14.0 ± 0.73 cm) was the longest, while the colon length shortening in the DSS group was significant (*p* < 0.0001). The colons were longer in the XSP groups with a length range of 12.16–19.95 cm. As expected, the change in colon shortening in the DSS group could be partly reversed by XSP supplementation.

### XSP Inhibits Alleviated Colonic Morphological Damage and Inflammatory Cell Infiltration in Ulcerative Colitis in Rats

**FIGURE 3:**
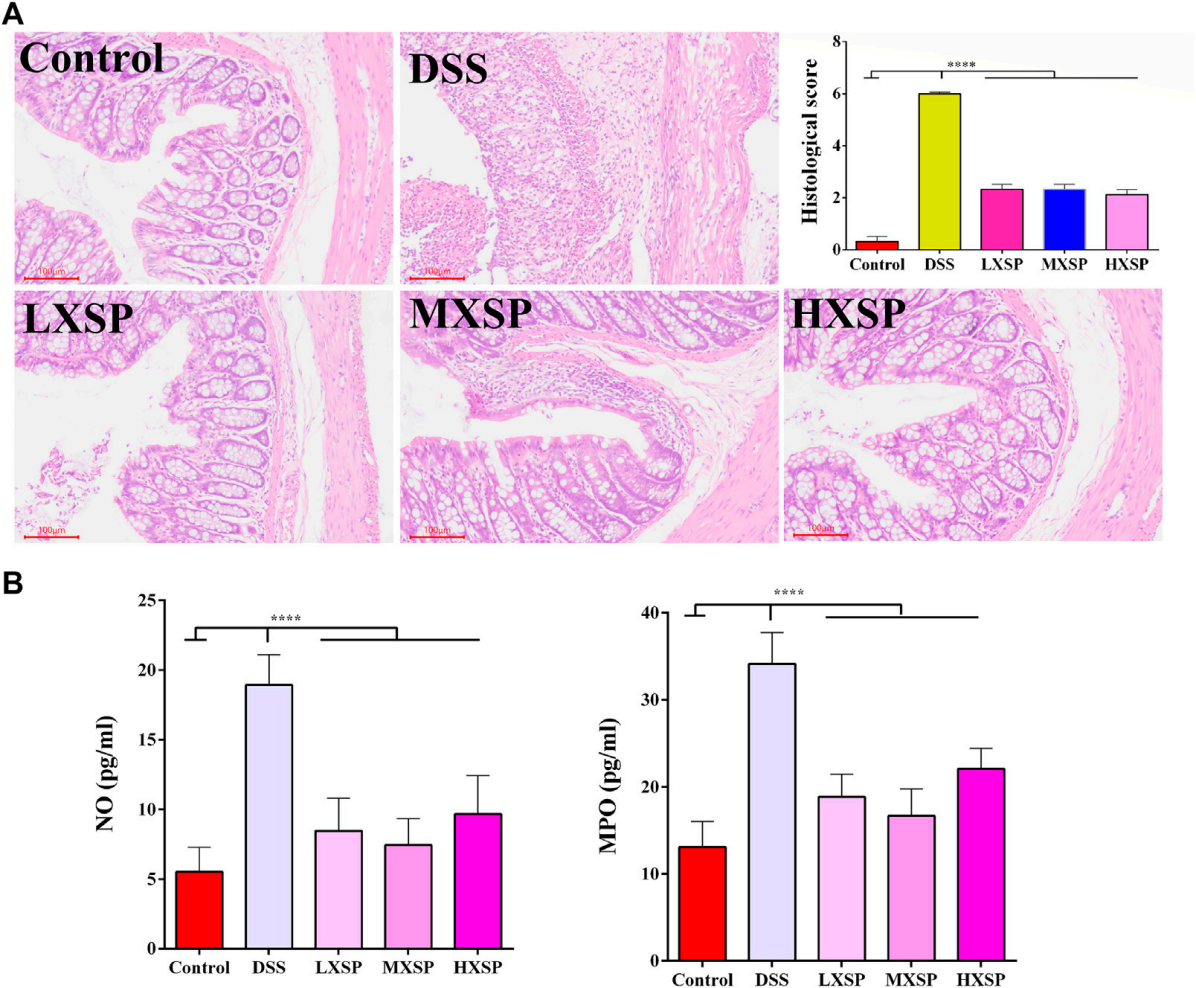
H&E staining of colon tissues and the activities of NO and MPO. **(A)** Epithelial damage, inflammatory cell infiltration, and crypt lesions in different groups were evaluated by H&E staining, and pathological scores were quantified in a bar graph. **(B)** Activities of MPO and NO were determined to indicate the degree of inflammation in colon tissue. Data are represented as mean ± SEM. *p < 0.05, **p < 0.01, ***p < 0.001, and ****p < 0.0001.

H&E staining in the colon tissues was performed to show the protective effect of XSP in colon injury. In the DSS group, the colon structure was seriously destroyed, and severe inflammatory cell infiltration was observed in the muscular layer of the colon. XPS treatment could significantly alleviate the colonic morphological damage caused by DSS. Reduced crypt epithelial aberrations and less inflammatory cell infiltration were also observed in XSP groups (Figure 3A; *p* < 0.0001). Furthermore, the activities of NO and MPO were measured to indicate the degree of inflammation in colon tissue. As shown in Figure 3B, colonic tissues in the DSS group showed increased levels of MPO and NO compared to the colonic tissues in the other groups. It was found that XSP in different doses remarkably reduced the increasing MPO and NO activities caused by DSS (Figure 3A; *p* < 0.0001).

### XSP Decreases the Expression of Pro-Inflammatory Cytokines in Ulcerative Colitis in Rats

**FIGURE 4:**
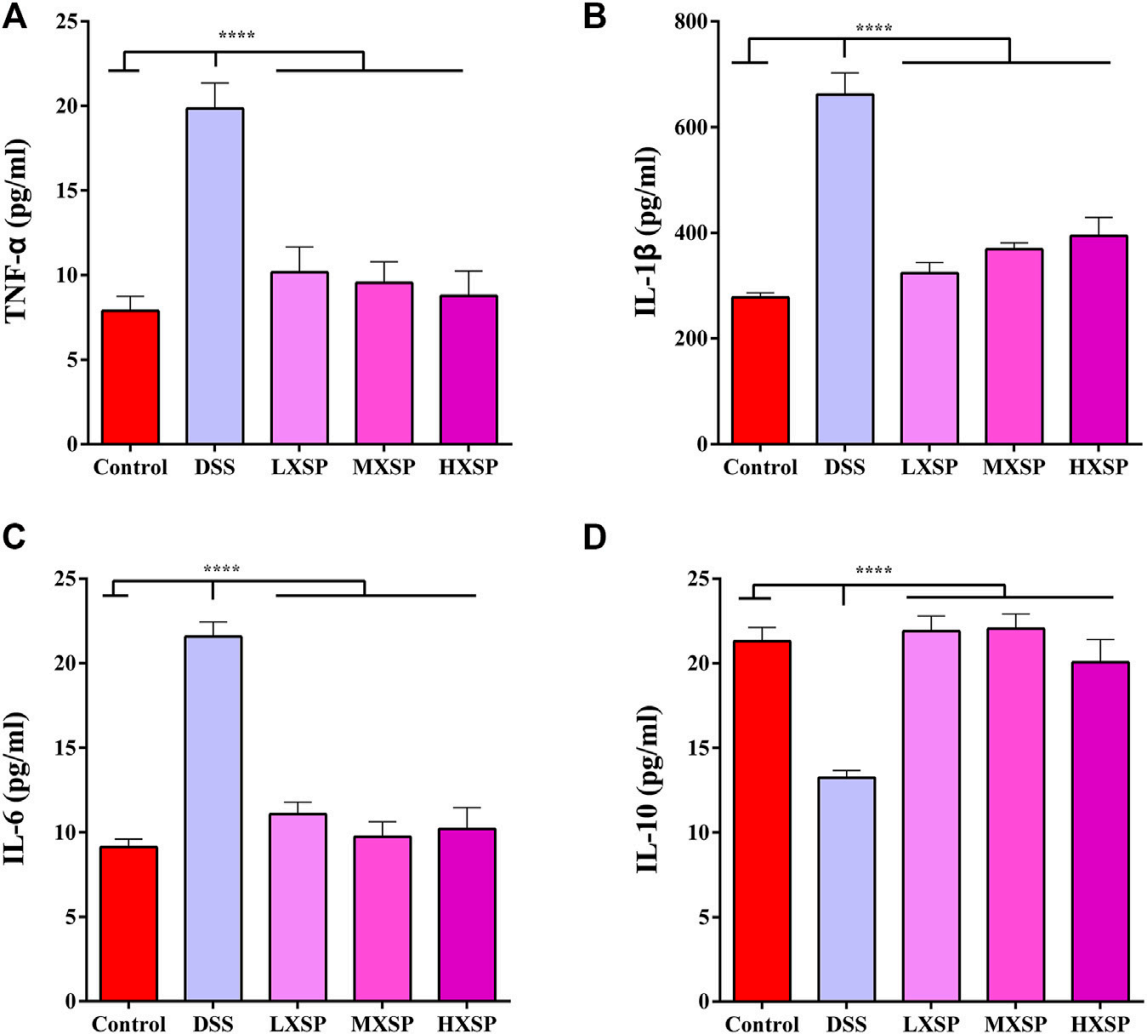
Protein levels of pro-inflammatory cytokines **(A)** TNF-α, **(B)** IL-β1, **(C)** IL-6, and **(D)** IL-10 in the serum of control, DSS, LXSP, MXSP, and HXSP groups. Data are represented as mean ± SEM. *p < 0.05, **p < 0.01, ***p < 0.001, and ****p < 0.0001.

As shown in Figures 4A–C, the protein levels of TNF-α, IL-1β, and IL-6 in the serum were significantly increased in the DSS group compared with the control group (*p* < 0.0001). However, XSP treatment dramatically decreased the levels of the inflammatory factors. In addition, XSP intervention significantly upregulated the protein level of IL-10 in UC in rats (*p* < 0.0001). Thus, the present results indicated that XSP could obstruct the inflammatory response, thus contributing to the treatment of UC.

### XSP Regulates the Imbalance of Gut Microbiota in Ulcerative Colitis in Rats

**FIGURE 5:**
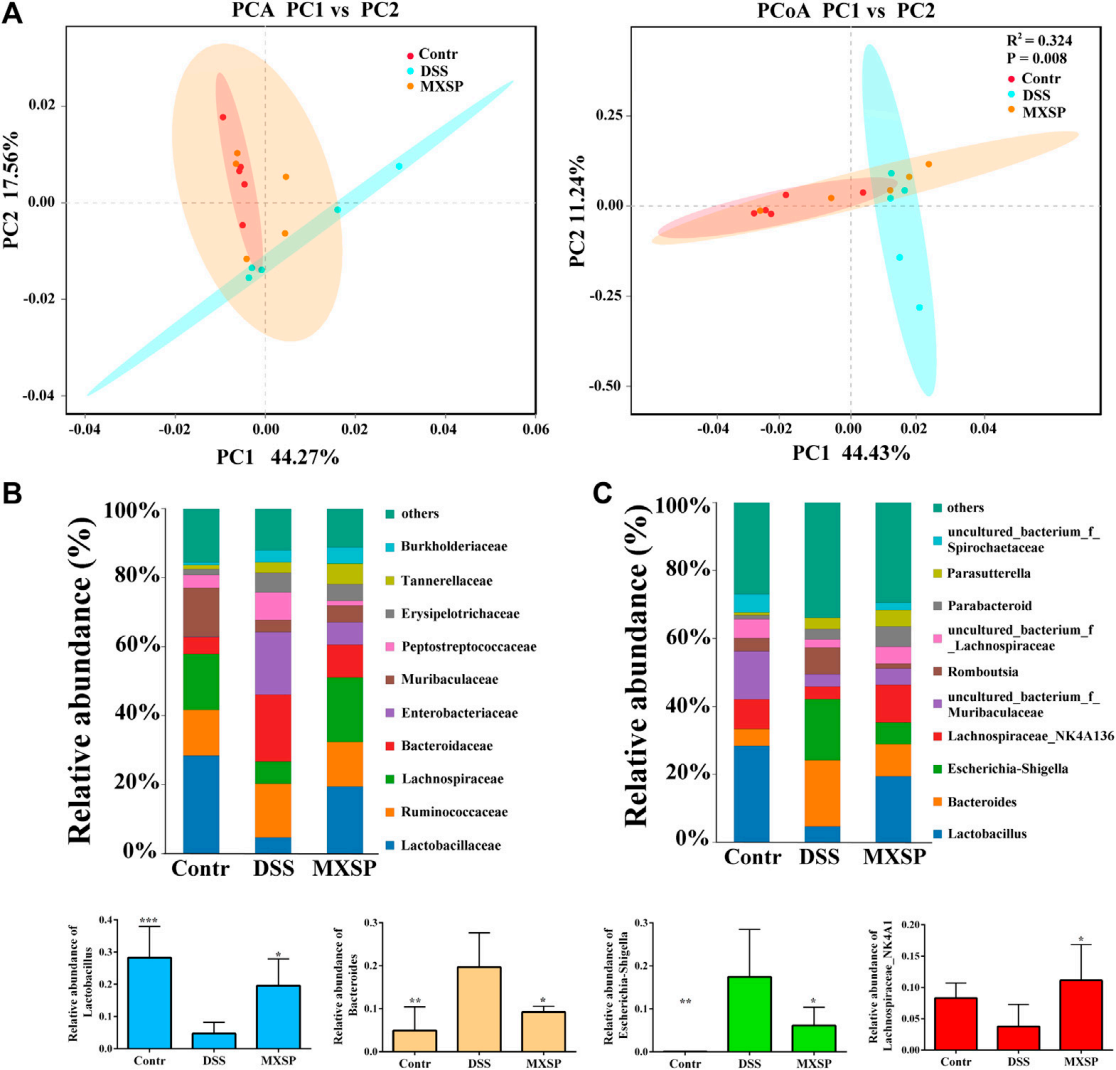
XSP reprograms gut microbiota and increases the abundance of SCFA-producing bacteria. **(A)** Principal component analysis (PCA) and principal coordinate analysis (PCoA) of gut microbial communities in control, DSS, and MXSP groups. **(B)** Fecal microbiota composition at the family level. **(C)** Fecal microbiota composition at the genus level and the proportions of the Lactobacillus, Bacteroidetes, *Escherichia–Shigella*, and the Lachnospiraceae_NK4A136 groups. Data are represented as mean ± SEM. *p < 0.05, **p < 0.01, ***p < 0.001, and ****p < 0.0001.

To further confirm the protective effects of XSP on the gut microbiota dysbiosis induced by DSS, fecal bacterial DNA was extracted and sequenced. Beta diversity analysis of the samples, including principal component analysis (PCA) and principal coordinate analysis (PCoA), was carried out using QIIME software. The results showed that gut microbiota in the DSS group were clearly different from those in the control and XSP groups. In addition, samples in the control and XSP groups were clustered together (Figure 5A). The relative species abundance and diversity of gut microbiota were reduced in DSS-induced UC in rats, while MXSP treatment could partly restore the balance in the microbiota community. As shown in Figures 5B,C, at the family level, decreased Lactobacillaceae, Lachnospiraceae, and Muribaculaceae content and increased Bacteroidaceae, Enterobacteriaceae, and Peptostreptococcaceae content in the DSS group was restored after MXSP intervention. At the genus level, the relative abundance of *Lactobacillus* and Lachnospiraceae_NK4A136 decreased in the DSS group but were remarkably elevated in the MXSP treatment group (*p* < 0.05). In contrast, DSS induced increased abundance of *Bacteroides* and *Escherichia–Shigella*, which were decreased by MXSP treatment. The present results showed that XSP administration could restore the gut microbiota dysbiosis to a healthy balance.

### XSP Modulates SCFA Production in Ulcerative Colitis in Rats

**FIGURE 6:**
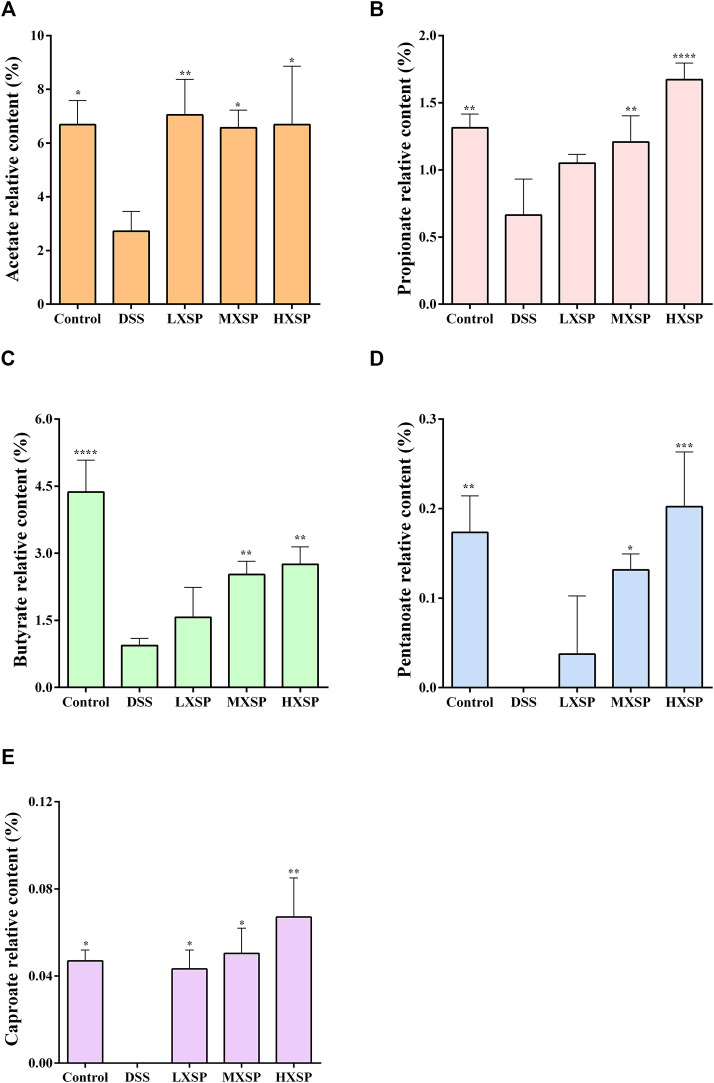
Relative levels of acetate **(A)**, propionate **(B)**, butyrate **(C)**, pentanoate **(D)**, and caproate **(E)** in fecal samples. Data are represented as mean ± SEM. *p < 0.05, **p < 0.01, ***p < 0.001, and ****p < 0.0001.

Previous research has indicated that the levels of SCFAs in UC patients were lower than those in non-UC individuals (Deleua et al., 2021). As the gut microbiota was changed in DSS-induced UC in rats, the varieties of SCFAs in rat feces were further analyzed by gas chromatography–mass spectrometry (GC-MS). As shown in Figure 6, five components of SCFAs were identified and quantified, including acetate, propionate, butyrate, pentanoate, and caproate. The results demonstrated that acetate, propionate, and butyrate represent a higher proportion of SCFAs, while the contents of pentanoate and caproate were very low. Compared to other groups, the levels of SCFAs in the DSS group significantly declined, and two components, pentanoate and caproate, were even undetectable (*p* < 0.05). Conversely, the levels of SCFAs considerably increased after XSP intervention (*p* < 0.05). In addition, the increase of SCFAs showed a dose-dependent trend. Thus, the present results demonstrated that XSP reversed the SCFA reduction in UC in rats induced by DSS.

### XSP Reduced Systemic Inflammation in Ulcerative Colitis in Rats

**FIGURE 7:**
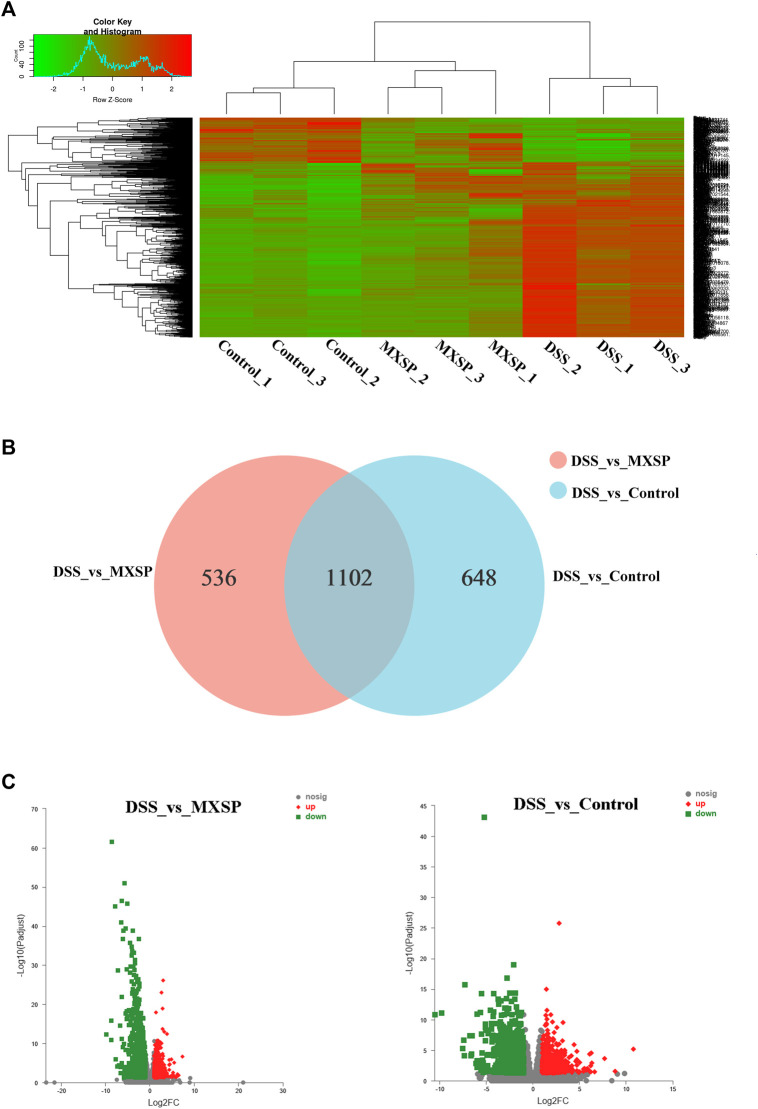
Differentially expressed genes (DEGs) in colonic tissue. **(A)** Heat map plot of DEGs in control, DSS, and MXSP groups. **(B)** Venn diagram of DEGs with DSS vs. control and DSS vs. MXSP. **(C)** Volcano plot of DEGs between DSS vs. control and DSS vs. MXSP in colonic tissue. Note: The black dots represent genes without differential expression between two groups, the red dots show genes that were upregulated, and the green dots show those that were downregulated.

To further explore the underlying mechanism of XSP on UC prevention, RNA-seq analysis of rat colons from control, DSS, and MXSP groups was carried out. The DEGs in the experimental groups were determined and analyzed (|log_2_FC|≥1 and *p* < 0.05). As shown in the heat map (Figure 7A), the DEGs in the DSS group showed less commonality with those in the XSP treatment group. Moreover, the samples in the MXSP and control groups formed a branch shown by using the hierarchical cluster analysis. Specifically, 1,750 DEGs in rat colonic tissue were identified after administration of 4% DSS, whereas 1,638 DEGs were identified in rat colonic tissue treated with MXSP. Overall, 617 upregulated and 1,021 downregulated DEGs were identified in the DSS vs. MXSP groups. There were 824 upregulated and 926 downregulated DEGs identified in the DSS vs. control groups. There were 1,102 DEG genes associated with the control, DSS, and MXSP groups (Figures 7B,C).

**FIGURE 8:**
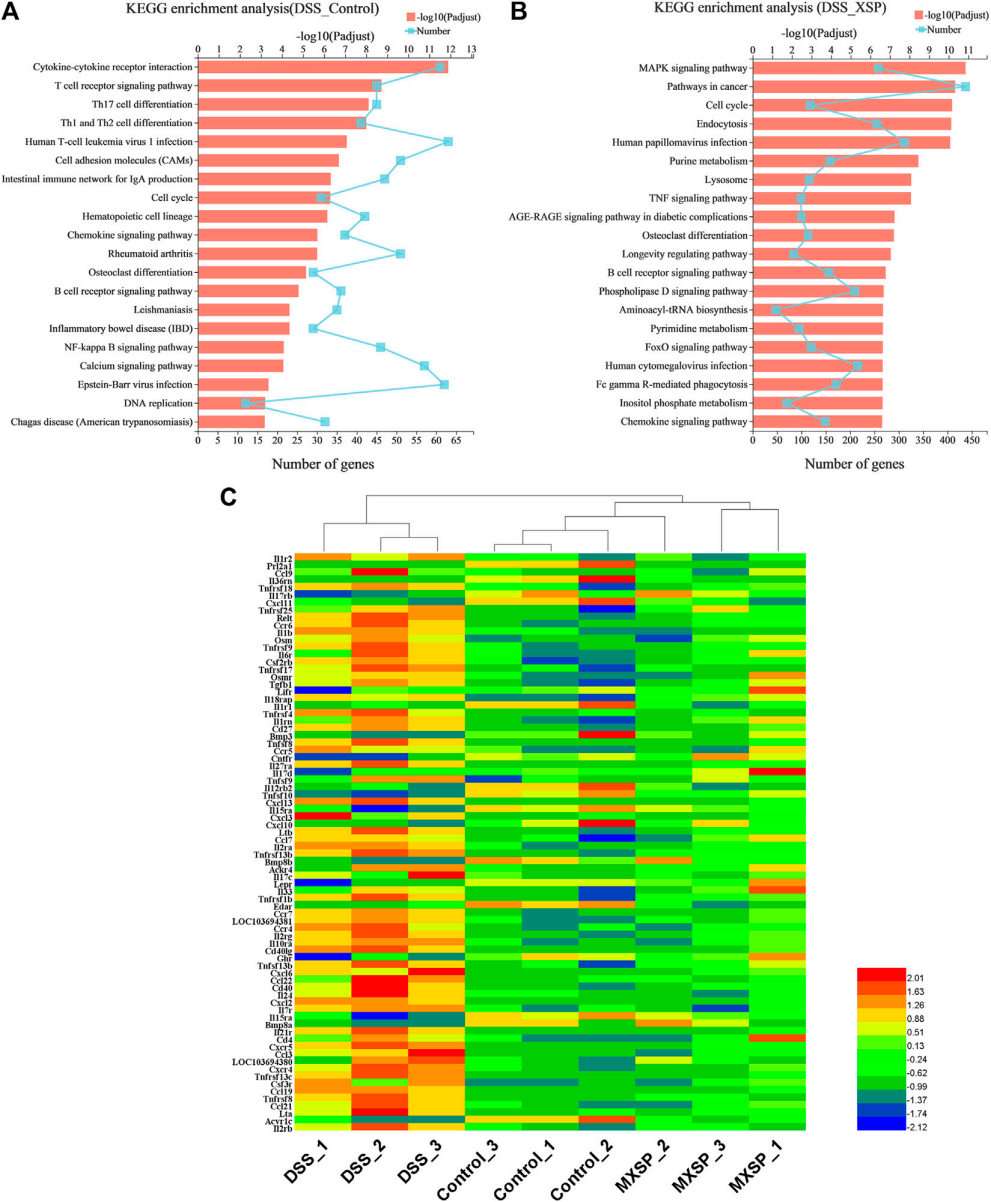
KEGG pathway analysis of DEGs. **(A)** Significant pathways involving DEGs between DSS and control groups. **(B)** Significant pathways involving DEGs between DSS and MXSP groups. **(C)** Heat map plot of genes in the MAPK signaling pathway.

The KEGG analysis was further analyzed to thoroughly investigate the potential pathways involved in the inflammatory responses. There were 80 and 93 pathways enriched in the DSS vs. control groups and DSS vs. MXSP groups, respectively (Supplementary Tables S1, S2; *p* < 0.05). The results revealed that pathways involved in signaling molecules, interaction (cytokine–cytokine receptor interaction and cell adhesion molecules), and the immune system (T-cell receptor signaling pathway, Th17 cell differentiation, Th1 and Th2 cell differentiation, intestinal immune network for IgA production, and hematopoietic cell lineage) were targeted in the DSS vs. control groups (Figure 8A). The pathways related to inflammation, cell cycle, and cancer were mainly enriched in the DSS vs. MXSP groups. The immune and inflammation-associated pathways included the MAPK signaling pathway, TNF signaling pathway, and B-cell receptor signaling pathway in the DSS vs. MXSP groups (Figure 8B). There were 78 candidate inflammatory genes targeted within the classic MAPK signaling pathway (map04060). These genes were further analyzed by Helm software for hierarchical cluster analysis. As shown in Figure 8C, the samples in the control and MXSP groups formed a branch, while the samples in the DSS group formed a single cluster. The expression levels of the inflammatory factors in the DSS group were clearly upregulated, while XSP treatment decreased this change.

## Discussion

Cucurbitacins, a class of triterpenoid compounds, which widely exist in Cucurbitaceae, are bitter and considered as specific insect attractants (Chambliss and Jones, 1966). Recent studies have demonstrated that cucurbitacins possess various bioactivities such as anti-inflammatory, antitumor, and antibacterial properties (Alghasham, 2013; Qiao et al., 2013; Kim et al., 2015; Ramezani et al., 2017). However, the pharmacological activity of cucurbitacins against UC in animals and the relative mechanisms are still less known. Thus, in the present study, we extracted and purified cucurbitacins from *Hemsleya* and prepared sustained release pellets with the extracted materials. Then, XSP was investigated in DSS-induced UC in rats to show its anti-inflammatory effect and the underlying mechanism. It was found that XSP intervention could improve the ongoing DSS-induced UC in rats. Compared to the DSS group, rats in the XSP groups had less frequent diarrhea and bloody stools. Meanwhile, XSP treatment could significantly reduce weight loss compared with the DSS-induced group. XSP treatment also ameliorated other symptoms of UC in rats, including low DAI score, reduced inflammatory cell infiltration in colon, and decreased expressions of pro-inflammatory cytokines. The results indicate that XSP administration impedes the inflammatory response in DSS-induced UC in rats. Furthermore, RNA transcriptome sequencing analysis revealed that XSP could suppress activation of the MAPK signaling pathway in UC in rats. The MAPK signaling pathway in mammals can involve various physiological activities including proliferation, apoptosis, inflammation, and inherent immunity (Kim et al., 2015). Thus, systematic inflammation in the XSP group could be diminished by impeding the MAPK signaling pathway.

Gut microbiota are considered a key factor in activating the immune system. The disturbance of the gut microbiota–host balance is associated with UC (Agus et al., 2018). In the present study, alterations in gut microbiota diversity and composition after XSP treatment were identified by fecal 16S rRNA sequencing. A dramatic decrease in microbiota diversity and composition of the DSS group was different from that of the control and XSP groups. The results from the beta diversity analysis showed that rat samples in the MXSP group were gathered into the control group and separated from the DSS group, which indicated that XSP intervention normalized the gut microbiota dysbiosis. The DSS-induced gut microbiota dysbiosis may be due to the decrease in symbiotic and increase in pathogenic bacteria. Our data showed that important SCFA producers Lactobacillaceae and Lachnospiraceae at the family level sharply declined in the DSS group but were elevated after XSP supplementation. The present study further demonstrated that the pathogenic family Bacteroideceae was the predominant bacteria in the DSS group, while the SCFA-producing family Lactobacillaceae had a high proportion in the control and XSP groups. At the genus level, the decreased abundance of *Lactobacillus* and the Lachnospiraceae_NK4A136 group induced by DSS could be significantly reversed to nearly normal levels by XSP treatment. The two genera of microbiota are beneficial bacteria and belong to the families of Lactobacillaceae and Lachnospiraceae, respectively. *Lactobacillus* is a probiotic and considered to be able to elevate acetate production (Wang et al., 2021). In addition, *Lactobacillus* has also shown potential activity in treating inflammatory bowel disease (Niel et al., 2002; Marteau, 2006; Qin et al., 2021). The Lachnospiraceae_NK4A136 group consists of important butyrate-producing bacteria (Tian et al., 2020). The abundance of *Escherichia–Shigella* and *Bacteroides* remarkably increased in UC in rats. *Escherichia–Shigella* has been identified as a kind of bacteria that promotes lung and gut inflammation (Liu et al., 2020; Cobo et al., 2021; Zhou et al., 2021). *Bacteroides* showed increased abundance in UC, which is highly associated with the occurrence and exacerbation of gut inflammation (Setoyama et al., 2003; Valguarnera and Wardenburg, 2020). However, the increase in the two pathogenic bacteria was significantly attenuated by XSP treatment.

The alternation of gut microbial composition brought corresponding changes in their metabolites. As crucial metabolites of gut microbiota, SCFAs have received increasing attention as an important factor in the regulation of the gut epithelium and immune system (Venegas et al., 2019). After DSS induction, the total levels of the five main SCFAs, including acetate, propionate, butyrate, pentanoate, and caproate, were significantly reduced but were elevated by XSP treatment. Among SCFAs, butyrate, in particular, is involved in the immune system (Yoo et al., 2020). Many studies have also revealed that butyrate has potential activity in relieving symptoms and inflammation of IBD (Deleua et al., 2021). The present results indicated that XSP administration significantly reversed the decrease in butyrate production caused by DSS. Coincidentally, the abundance of the butyrate-producing bacteria Lachnospiraceae_NK4A136 group in Lachnospiraceae family was greatly elevated after XSP intervention, showing positive correlation between butyrate and its producer. In addition, another increased beneficial microbiota, *Lactobacillus*, was also positively related to elevation of acetate production in the XSP group. As a result, the elevation of SCFA levels after XSP administration was caused by the alterations of microbial composition, especially the increased production of the Lachnospiraceae_NK4A136 group and *Lactobacillus*. Moreover, the elevation of SCFAs in XSP groups occurred in a dose-dependent manner. Meanwhile, the inflammatory state of UC in rats was lessened by XSP intervention but was not totally dependent on the dosages of XSP. The increased SCFAs and decreased inflammatory symptoms with XSP intervention showed a negative correlation, although the trend with different doses is not exactly consistent. This inconsistency may be due to preventive XSP treatment as SCFA production was directly reversed by maintaining the microbiota composition during development of inflammation. Inflammation results from a combination of many factors and is a complex pathological process. In addition, it was difficult to accurately evaluate the specific role of individual or mixed SCFAs on anti-UC (Ran An et al., 2019). Moreover, whether XSP has an anti-inflammatory effect independent of gut microbiota also needs to be further studied. SCFAs are considered mediators of gut bacteria and inflammation, and exogenous SCFA administration has been suggested as an effective remedy to ease inflammation (Gill et al., 2018). SCFAs were reported to control the expression of inflammatory cytokines in modulating protective immunity and tissue inflammation. This modulation was achieved by regulating the MAPK signaling pathway and promoting GPR41 and GPR43 expression (Kim et al., 2013). Coincidentally, the results from colonic RNA-seq analysis targeted only the MAPK signaling pathway. It was found that the expression of genes related to inflammation in the MAPK signaling pathway was suppressed after XSP intervention, resulting in a reduction in tissue inflammation. Therefore, SCFAs resisted intestinal inflammation probably *via* modulating MAPK signaling pathways in this study. However, further precise mechanisms need to be identified.

## Conclusion

The present study showed that XSP exhibited protective effects against DSS-induced UC in rats. The anti-inflammatory effect of XSP was dependent on its potential ability to restore gut microbiota balance and increase SCFA production. The enhanced SCFAs ameliorated colonic inflammation *via* suppressing the MAPK signaling pathway. These findings offer support for the potential application of XSP in prevention of UC (Kim and Choi, 2015).

## Data Availability

The datasets presented in this study can be found in online repositories. The names of the repository/repositories and accession number(s) can be found below: NCBI under accession PRJNA798167 for data of colonic RNA-seq and PRJNA796647 for 16S rRNA data.
